# Mid-Term Outcomes After Coronary Artery Bypass Grafting and the Impact of Glucose Metabolism Disorders on Prognosis

**DOI:** 10.3390/jcm15218526

**Published:** 2026-11-02

**Authors:** Alexey N. Sumin, Natalia A. Bezdenezhnykh, Olga V. Gruzdeva, Olga L. Barbarash

**Affiliations:** 1 Federal State Budgetary Scientific Institution “Research Institute for Complex Issues of Cardiovascular Diseases”, Academician L.S. Barbarash Boulevard, 6, Kemerovo 650002, Russia; o_gruzdeva@mail.ru (O.V.G.); olb61@mail.ru (O.L.B.); 2 Federal State Budgetary Educational Institution of Higher Education, Kemerovo State Medical University, Voroshilova st., 22A, Kemerovo 650056, Russia

**Keywords:** metabolism disorders, MACE, mid-term outcomes, prediabetes, type 2 diabetes mellitus, glucose

## Abstract

**Objective**: To assess the relationship between preoperative glycemic status (normoglycemia, prediabetes, and overt type 2 diabetes mellitus) and mid-term (4-year) outcomes after coronary artery bypass grafting (CABG). **Methods**: The analysis included 693 patients who underwent elective CABG between 1 May 2016 and 1 May 2018 and had known glycemic status. The median follow-up among survivors was 50 months (4.2 years; range 29–73 months). Based on preoperative glycemic status, actively diagnosed using an oral glucose tolerance test, patients were divided into three groups: normoglycemia (*n* = 293), prediabetes (*n* = 229), and type 2 diabetes mellitus (T2DM, *n* = 171). Major adverse cardiovascular events (MACE) were defined as death from any cause, myocardial infarction, or stroke. Kaplan–Meier analysis, logistic regression, and Cox proportional hazards regression were used to assess predictors of adverse outcomes. **Results**: A clear gradient in adverse event rates emerged across the three glycemic categories: lowest in normoglycemia, intermediate in prediabetes, and highest in T2DM. All-cause mortality was 10.6% in normoglycemia, 15.3% in prediabetes, and 28.7% in T2DM (*p* < 0.001 for Groups 1–3; *p* = 0.001 for Groups 2–3). MACE occurred in 15.7%, 24.5%, and 39.2%, respectively (*p* < 0.001). Prediabetes was associated with a higher incidence of MACE than normoglycemia (24.5% vs. 15.7%; *p* = 0.002) but not with higher mortality. Kaplan–Meier analysis showed significantly lower survival in patients with any glucose metabolism disorder (*p* = 0.002) and in those with T2DM (*p* < 0.001) compared with normoglycemia; freedom from MACE was significantly higher in normoglycemia than in T2DM (*p* < 0.001), prediabetes (*p* = 0.007), and any glucose metabolism disorder (*p* < 0.001). In multivariable logistic regression, T2DM was independently associated with MACE after adjustment for sex, age, combined procedures, and eGFR (OR 1.59, 95% CI 1.33–1.90; *p* < 0.001); prediabetes remained independently associated after adjustment for sex, age, combined procedures, CPB duration, and eGFR (OR 1.21, 95% CI 1.05–1.40; *p* = 0.009); any glucose metabolism disorder was associated with MACE after adjustment for sex, age, combined procedures, and eGFR (OR 1.31, 95% CI 1.16–1.48; *p* < 0.001). In Cox proportional hazards regression, independent predictors of mid-term mortality and MACE were decreased renal function (*p* = 0.046 and *p* = 0.037, respectively), aortic cross-clamp time (*p* < 0.001 for both), and the presence of any glucose metabolism disorder (*p* < 0.001 for both). **Conclusions**: Preoperative glucose metabolism disorders were associated with worse mid-term (4-year) outcomes after CABG. T2DM was associated with higher all-cause mortality, whereas prediabetes was associated with a higher incidence of MACE but not with mortality. These observational findings support the clinical relevance of identifying and managing prediabetes before CABG, although causal effects cannot be inferred.

## 1. Introduction

Diabetes mellitus (DM) is one of the risk factors for the development and progression of atherosclerosis [1,2]. When managing patients with coronary artery disease (CAD), the coexistence of diabetes mellitus often alters the choice of revascularization method. In such cases, open heart surgery tends to be preferred over catheter-based interventions [3,4]. Moreover, type 2 DM exerts a notable influence on two key aspects of CABG outcomes: the immediate perioperative period [5] and the patient’s longer-term clinical trajectory following the procedure [6]. The strategic direction of recent years is not only taking into account the presence of DM in the treatment of cardiac patients, but also its active detection in cardiovascular diseases [1]. Based on this, in the CABG preoperative period, it is proposed to identify not only type 2 DM, but also other disorders of glucose metabolism, which are usually classified as prediabetes. The studies conducted have not yet provided a definitive answer to the advisability of identifying prediabetes in this category of patients.

A recently published study by our research group [7] showed that with active preoperative screening, newly diagnosed type 2 DM was detected in 6.9% of patients, and prediabetes in 29% of patients. Both the presence of type 2 DM and the presence of prediabetes were associated with an increased incidence of perioperative complications (mainly cardiovascular and surgical wound complications). However, studies on the effect of prediabetes on long-term prognosis after myocardial revascularization have shown conflicting results [8,9,10,11]. Accordingly, the question of whether the long-term impact of prediabetes on outcomes depends on the method of myocardial revascularization in patients with CAD remains open.

## 2. Material and Methods

### 2.1. Study Design

This investigation examined the mid-term outcomes of individuals undergoing elective or priority elective coronary artery bypass grafting (CABG) between 1 May 2016 and 1 May 2018. In the present study, elective CABG was defined as surgery performed after a standard waiting period in clinically stable patients. Priority elective CABG referred to surgery scheduled earlier than usual due to clinical priority (e.g., symptom progression or high-risk anatomy) but not meeting criteria for urgent or emergency intervention. A study cohort with well-characterized glycemic status was established at the time of the initial preoperative assessment. Beyond the standard preoperative workup, subjects without a prior diagnosis of diabetes mellitus underwent testing for latent glucose metabolism disorders, specifically undiagnosed diabetes mellitus or prediabetes. Comprehensive details regarding cohort assembly, baseline patient demographics, perioperative glucose management strategies, and preoperative diagnostic data—including the identification of glucose metabolism disorders—have been published previously by our team [7]. The study protocol was approved by the local ethics committee at the Research Institute for Complex Issues of Cardiovascular Diseases (protocol No. 20160421, approved 21 April 2016).

### 2.2. Study Population

The original cohort comprised 1021 patients who underwent CABG between 1 May 2016 and 1 May 2018 and had known glycemic status. These patients represent a subset (68%) of a previously described cohort from our research group (*n* = 1021); the defining feature of this cohort was universal preoperative screening for glucose metabolism disorders [7]. A detailed flow diagram of cohort formation has been published previously [7].

Mid-term follow-up data were collected in several sequential phases. First, the entire cohort (*n* = 1021) was audited using the institutional electronic medical record system to identify all clinic visits, hospital admissions, and deaths—including postoperative deaths—that occurred during the study period. Second, for patients with unknown outcomes, official inquiries were submitted to the civil registry office, and the obtained information was entered into the registry; this step allowed us to ascertain a substantial proportion of deaths and their causes. Third, letters containing the contact details of the study physician and a request to contact the research team were sent to all remaining addresses; information was then collected during return telephone calls and verified through the medical information system and other available sources. All available records of interventions, hospital admissions, examinations, and causes of death were collected and used to complete the primary documentation and the study database.

As a result, information sufficient for analysis was obtained for 693 patients, corresponding to a follow-up coverage of 68% of the original cohort. The median follow-up among survivors was 50 months (4.2 years) from the date of CABG [lower quartile Q1 45.0; upper quartile Q2 60.0]. The shortest follow-up was 29 months and the longest was 73 months from the date of CABG. For comparative analyses, patients were stratified into three categories according to preoperative glycemic status: Group 1—normal glucose metabolism (normoglycemia); Group 2—prediabetes; and Group 3—established type 2 diabetes mellitus (T2DM). The diagnostic criteria for glucose metabolism disorders have been reported in detail elsewhere [7].

### 2.3. Diagnosis of Glucose Metabolism Disorders

**Figure 1:**
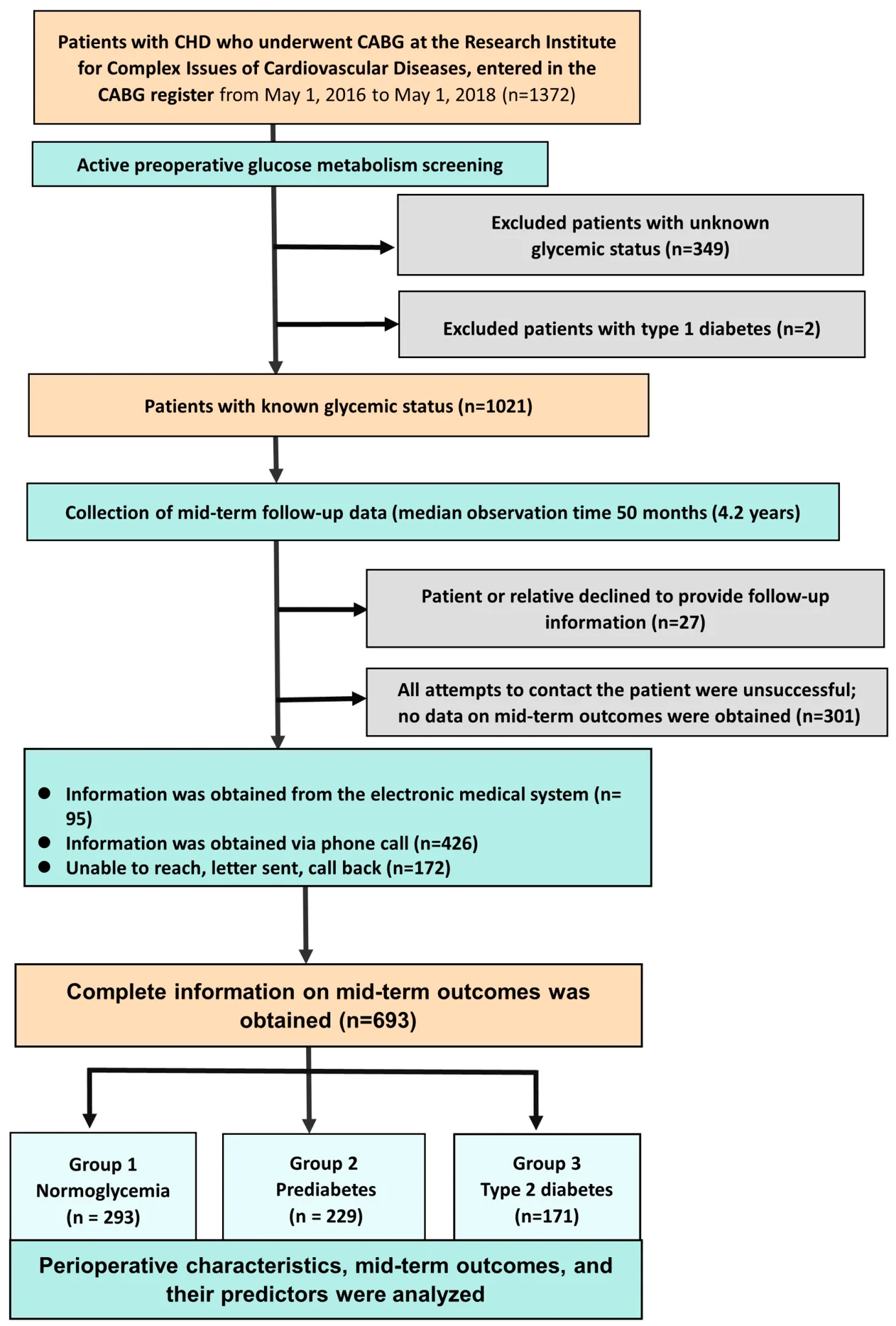
Study flow diagram. Notes: CABG—coronary artery bypass grafting; CHD—coronary heart disease. Patients were screened for glucose metabolism status preoperatively. After exclusions, 693 patients with available mid-term outcome data were stratified into three groups: Group 1 Normoglycemia (*n* = 293), Group 2 Prediabetes (*n* = 229), and Group 3 Type 2 diabetes (*n* = 171).

Diabetes mellitus and prediabetes were diagnosed using the World Health Organization (WHO) diagnostic criteria [12]. These criteria are consistent with those of the International Diabetes Federation (IDF) and the European Association for the Study of Diabetes (EASD), but differ from the American Diabetes Association (ADA) criteria with respect to the definition of prediabetes [12,13,14,15]. In the study sample, two patients with type 1 diabetes mellitus were excluded at the initial screening stage (Figure 1). No patients with other types of diabetes (other than type 1 or type 2) were present in the cohort. Therefore, throughout the remainder of this article, the terms “diabetes mellitus” and “diabetes” refer to T2DM unless otherwise specified.

Diabetes mellitus was diagnosed when one of the following parameters was detected: fasting blood glucose ≥ 7.0 mmol/L (126 mg/dL); blood glucose ≥ 11.1 mmol/L (200 mg/dL) 120 min after the oral glucose tolerance test (OGTT); or random blood glucose ≥ 11.1 mmol/L in the presence of characteristic symptoms of hyperglycemia (polydipsia, polyuria, general weakness). In the absence of acute metabolic decompensation, the diagnosis of diabetes mellitus was confirmed by two blood glucose measurements within the diabetic range, or by a single glycated hemoglobin (HbA1c) level ≥ 6.5% combined with a single blood glucose measurement [1,2].

To identify prediabetes (impaired fasting glucose [IFG] and/or impaired glucose tolerance [IGT]), the WHO criteria (1999–2013), which are also used in the Russian Federation, were applied, in contrast to the stricter thresholds proposed by the ADA. According to WHO recommendations, IGT was diagnosed when fasting plasma glucose was <7.0 mmol/L (126 mg/dL) and plasma glucose 2 h after a 75-g OGTT was 7.8–11.1 mmol/L (140–200 mg/dL). IFG was defined as fasting plasma glucose 6.1–6.9 mmol/L (110–125 mg/dL) and, when available, a 2-h post-OGTT glucose level < 7.8 mmol/L (<140 mg/dL) [15,16]. HbA1c < 6.0% was considered normal, and values of 6.0–6.4% corresponded to prediabetes. Prediabetes was defined as the presence of IFG, IGT, or a combination of both. A detailed description of the diagnostic algorithm, including the use of a computer program developed by the authors, has been published in our earlier article [7].

### 2.4. Follow-Up Outcomes

Major adverse cardiovascular events (MACE) were defined as death from any cause, myocardial infarction (MI), or stroke. All strokes, MIs, and deaths that occurred during the observation period—including those occurring postoperatively—were counted. Beyond MACE, the following were also recorded as cardiovascular events (CVE): repeat myocardial revascularization, non-coronary arterial surgery, amputations necessitated by peripheral atherosclerosis, emergency hospitalizations for cardiovascular disease (including hospitalization for acute coronary syndrome, decompensated chronic heart failure, and other cardiovascular causes), and recurrence of angina. All adverse events included, in addition to the above CVE, hospitalization for any cause, any surgical procedure, and COVID-19 during follow-up (including COVID-19 requiring hospitalization). An event was defined as the occurrence of at least one of these outcomes during follow-up after CABG.

### 2.5. Statistical Analysis

Factors associated with survival and the incidence of MACE were analyzed using both logistic regression (for binary outcomes) and Cox proportional hazards regression (for time-to-event outcomes). The outcome (MACE) and glucose status were coded as binary variables (MACE: 1 = event, 0 = no event; T2DM, prediabetes, or any glucose metabolism disorder: 1 = present, 0 = absent). No ordinal scores or per-unit variables were used.

Covariates were chosen a priori on the basis of clinical relevance and established prognostic factors; univariable analysis served only as a preliminary screening step. Logistic regression models were constructed to identify independent predictors of MACE, adjusting for clinically relevant covariates (sex, age, body mass index, left ventricular ejection fraction, left ventricular dimensions, left atrial dimension, combined procedures, cardiopulmonary bypass duration, aortic cross-clamp time, and estimated glomerular filtration rate, as appropriate for each model). Continuous covariates were entered linearly. To avoid collinearity, variables with a Pearson correlation coefficient > 0.7 were not included in the same model but were instead tested in separate models as a sensitivity analysis. All covariates were entered simultaneously (not stepwise). In these sensitivity analyses, glucose status was entered either as T2DM versus normoglycemia (with prediabetes excluded), as prediabetes versus normoglycemia (with T2DM excluded), or as any glucose metabolism disorder versus normoglycemia. Odds ratios (ORs) with 95% confidence intervals (CIs) are reported.

Missing data were handled by complete-case analysis (listwise deletion): patients with missing values for any variable included in a given model were excluded from that specific analysis, and no imputation (e.g., mean substitution) was performed. The threshold for statistical significance in regression analyses was set at *p* = 0.05 (two-sided).

The pre-specified multivariable Cox models included the following variables: age, sex, EuroSCORE II score, estimated glomerular filtration rate, aortic cross-clamp time, number of shunts, combined operations, total operative time, and any glucose metabolism disorder. Covariates were entered into the model without stepwise selection or preliminary univariable analysis. Model diagnostics, based on correlation analysis, revealed no significant multicollinearity. The number of events (outcomes) was deemed sufficient in relation to the number of model parameters (at least 10 events per predictor). The proportional hazards assumption was checked using Schoenfeld residuals; no violation was detected (all *p* > 0.05). For off-pump procedures, aortic cross-clamp time was coded as 0.

## 3. Results

During the 4.2-year follow-up after CABG, MACE occurred in 169 patients (24.4%), death from any cause in 119 patients (17.2%), and any cardiovascular event in 254 patients (36.7%). The distribution of adverse events by glycemic status is presented in Table 1. A clear gradient was observed across the three groups: patients with normoglycemia had the lowest event rates, those with prediabetes showed intermediate values, and patients with T2DM had the highest rates. All adverse events occurred in 125 (42.6%), 125 (54.4%), and 114 (66.5%) patients, respectively (*p* < 0.001). MACE occurred in 46 (15.7%), 56 (24.5%), and 67 (39.2%) patients (*p* < 0.001); pairwise comparisons were significant for Groups 1–3 (*p* < 0.001) and Groups 1–2 (*p* = 0.002). All-cause mortality was 31 (10.6%), 35 (15.3%), and 49 (28.7%) (*p* < 0.001; Groups 1–3 *p* < 0.001; Groups 2–3 *p* = 0.001). Cardiovascular mortality was 17 (5.8%), 21 (9.1%), and 30 (17.5%) (*p* < 0.001). Myocardial infarction occurred in 15 (5.2%), 17 (7.5%), and 22 (12.9%) patients (*p* = 0.011; Groups 1–3 *p* = 0.005). The incidence of stroke, percutaneous coronary intervention, carotid and lower-extremity arterial interventions, recurrent angina, and emergency hospitalizations for cardiovascular disease did not differ significantly between groups (all *p* > 0.05).

**Figure 2:**
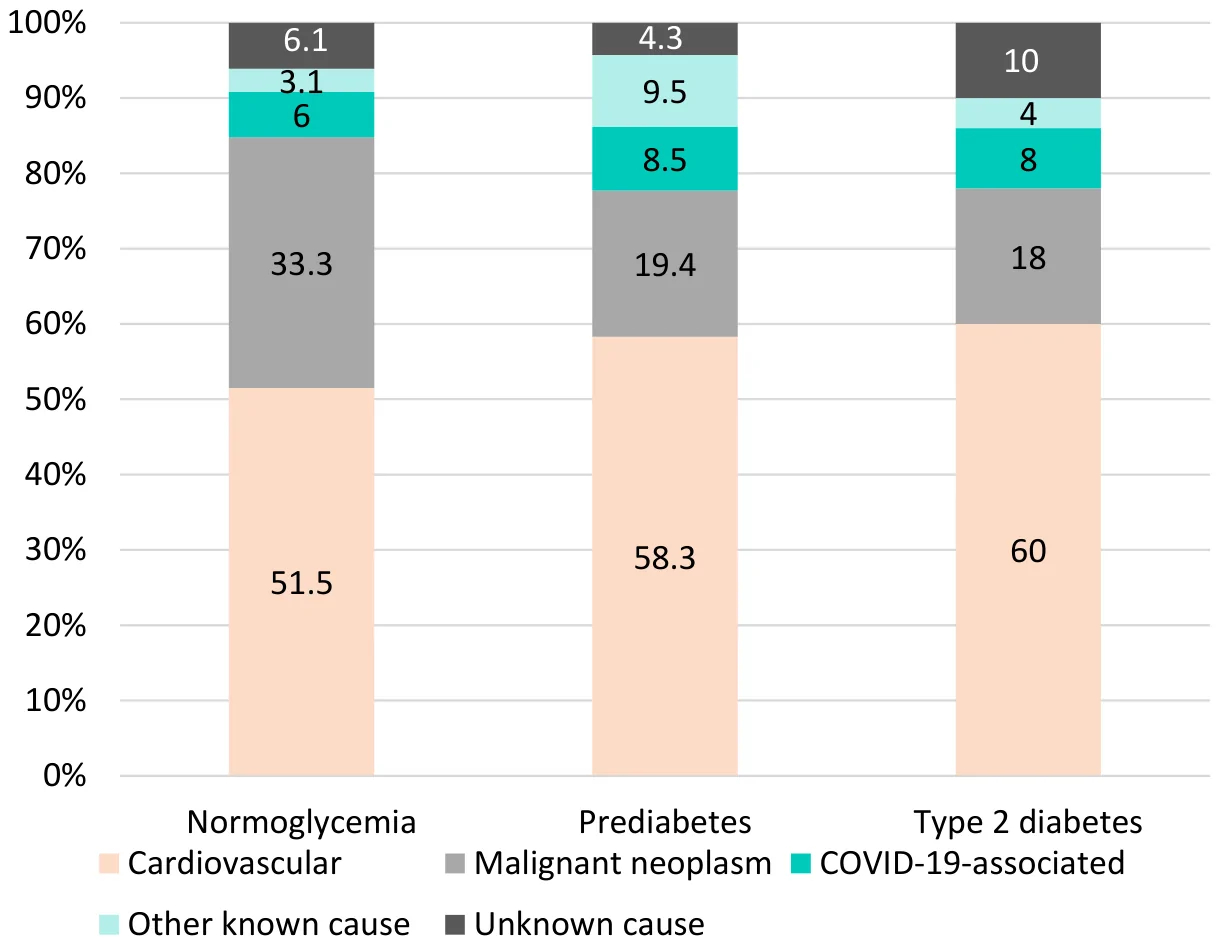
Causes of death after CABG during follow-up, stratified by glucose metabolism status.

The breakdown of mortality causes is shown in Figure 2, with no significant between-group differences observed. Cardiovascular disease was the predominant cause of death across all three groups, accounting for 52% of deaths in the normoglycemia group, 58% in the prediabetes group, and 60% in the diabetes group. The frequency of specific causes did not differ significantly among the groups. Malignancies ranked as the second most frequent cause of death in all three cohorts. Fatalities attributed to COVID-19 ranged from 6% to 8% across the groups, with no statistically significant differences.

The lowest number of all-cause hospitalizations was observed in the normoglycemic group (*p* = 0.008 vs. the prediabetes group; *p* = 0.013 vs. the diabetes group). Patients with diabetes and prediabetes tended to have more frequent hospitalizations for decompensated chronic heart failure (CHF) and acute coronary syndrome (ACS) compared with the normoglycemic group.

Baseline clinical characteristics are presented in Table 2. After Bonferroni correction (*p* < 0.017), significant differences between the three groups were observed for sex, age, body mass index (BMI), duration of hypertension, duration of coronary artery disease, and EuroSCORE II. In the T2DM group, compared with normoglycemia, there was a higher proportion of women (*p* < 0.001), older age (*p* = 0.006), higher BMI (*p* < 0.001), longer duration of hypertension (*p* < 0.001), longer duration of CAD (*p* = 0.003), and higher EuroSCORE II (*p* = 0.003). Between normoglycemia and prediabetes, significant differences were found for BMI (*p* < 0.001) and duration of hypertension (*p* < 0.001). Differences between prediabetes and T2DM in duration of hypertension (*p* = 0.042) and EuroSCORE II (*p* = 0.021) did not reach significance after adjustment.

Intraoperative and perioperative characteristics are presented in Table 3. The proportion of on-pump surgery was numerically highest in the prediabetes group (88.2%), but the difference across the three groups was not statistically significant (*p* = 0.398). Prediabetic patients had a longer duration of mechanical ventilation than normoglycemic patients (*p* = 0.001). All other surgical parameters were comparable across the three groups.

With regard to discharge pharmacotherapy, the prescription rate for aldosterone antagonists differed significantly across groups (*p* = 0.016), being higher among patients with type 2 diabetes (54.4) than among normoglycemic (46.1%; *p* = 0.008) and prediabetic (41.0%; *p* = 0.009) individuals. Although the overall *p*-value for statin prescription was 0.047, no pairwise comparison attained statistical significance after Bonferroni adjustment (α = 0.017), with the largest difference observed between prediabetic and normoglycemic patients (*p* = 0.027). No other between-group differences were detected for the remaining medication classes (*p* > 0.05 for all).

Coronary angiography revealed significant intergroup differences (*p* < 0.05) (Table 4). After Bonferroni correction (α = 0.0167), single-vessel disease was more frequently observed in normoglycemia and prediabetes than in T2DM; two-vessel disease was more prevalent in prediabetes than in both other groups; three-vessel disease was more prevalent in prediabetes than in normoglycemia. No significant intergroup differences were found in echocardiographic parameters, carotid artery stenosis, or lower-extremity artery stenosis.

Table 5 presents preoperative laboratory values by glycemic status. Fasting glucose and glycated hemoglobin showed a clear stepwise increase from normoglycemia to prediabetes and further to type 2 diabetes mellitus (both *p* < 0.001; all pairwise comparisons significant). Total cholesterol, low-density lipoprotein cholesterol, creatinine, urea, estimated glomerular filtration rate, activated partial thromboplastin time, fibrinogen, and soluble fibrin monomer complexes did not differ significantly between groups (all *p* > 0.05). High-density lipoprotein cholesterol was slightly lower in type 2 diabetes mellitus (*p* = 0.030), and triglycerides were higher in type 2 diabetes mellitus than in normoglycemia (*p* = 0.001; pairwise comparison between groups 1 and 3 *p* < 0.001). Overall, apart from the expected differences in glycemic parameters and triglycerides, the three groups were comparable with respect to lipid profile, renal function, and inflammatory markers.

**Figure 3:**
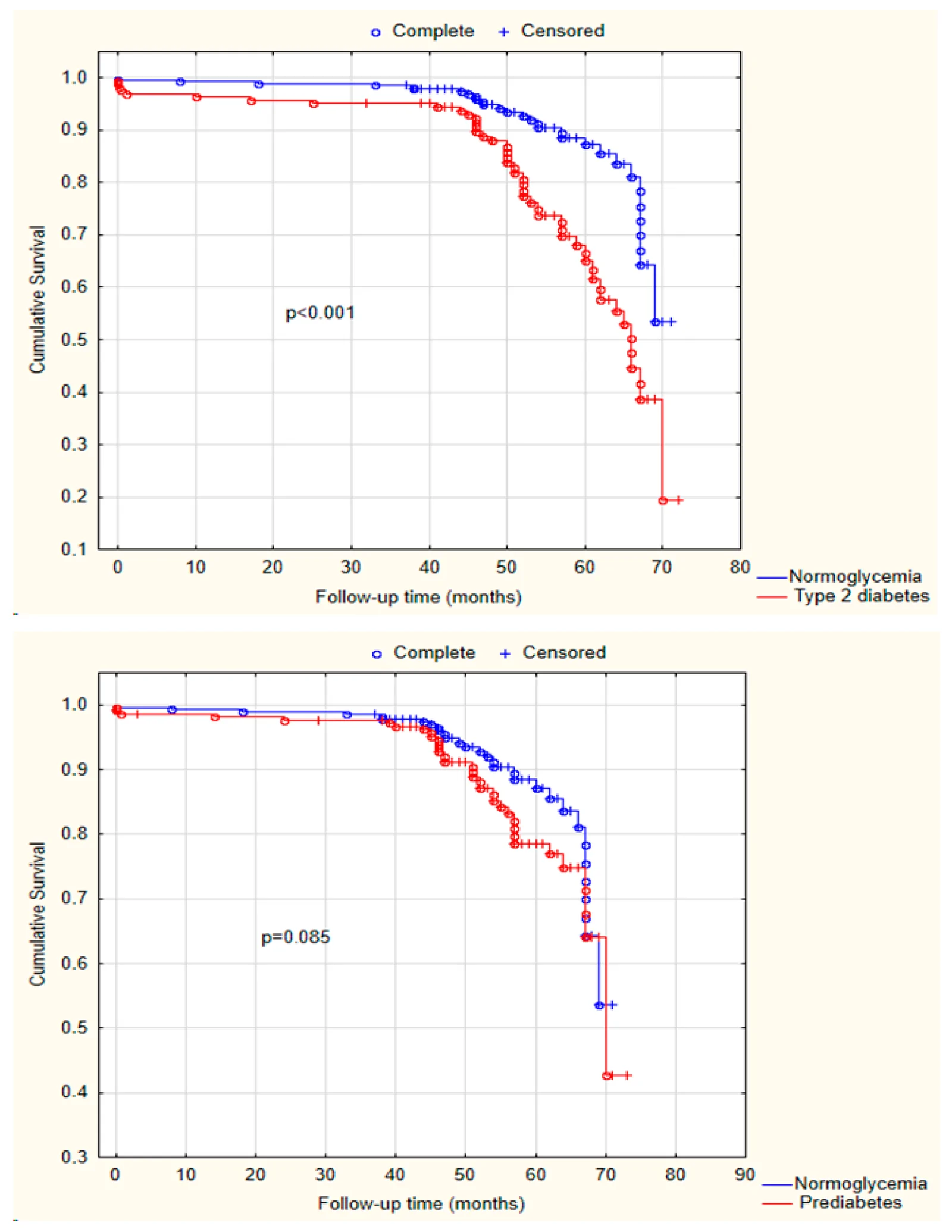

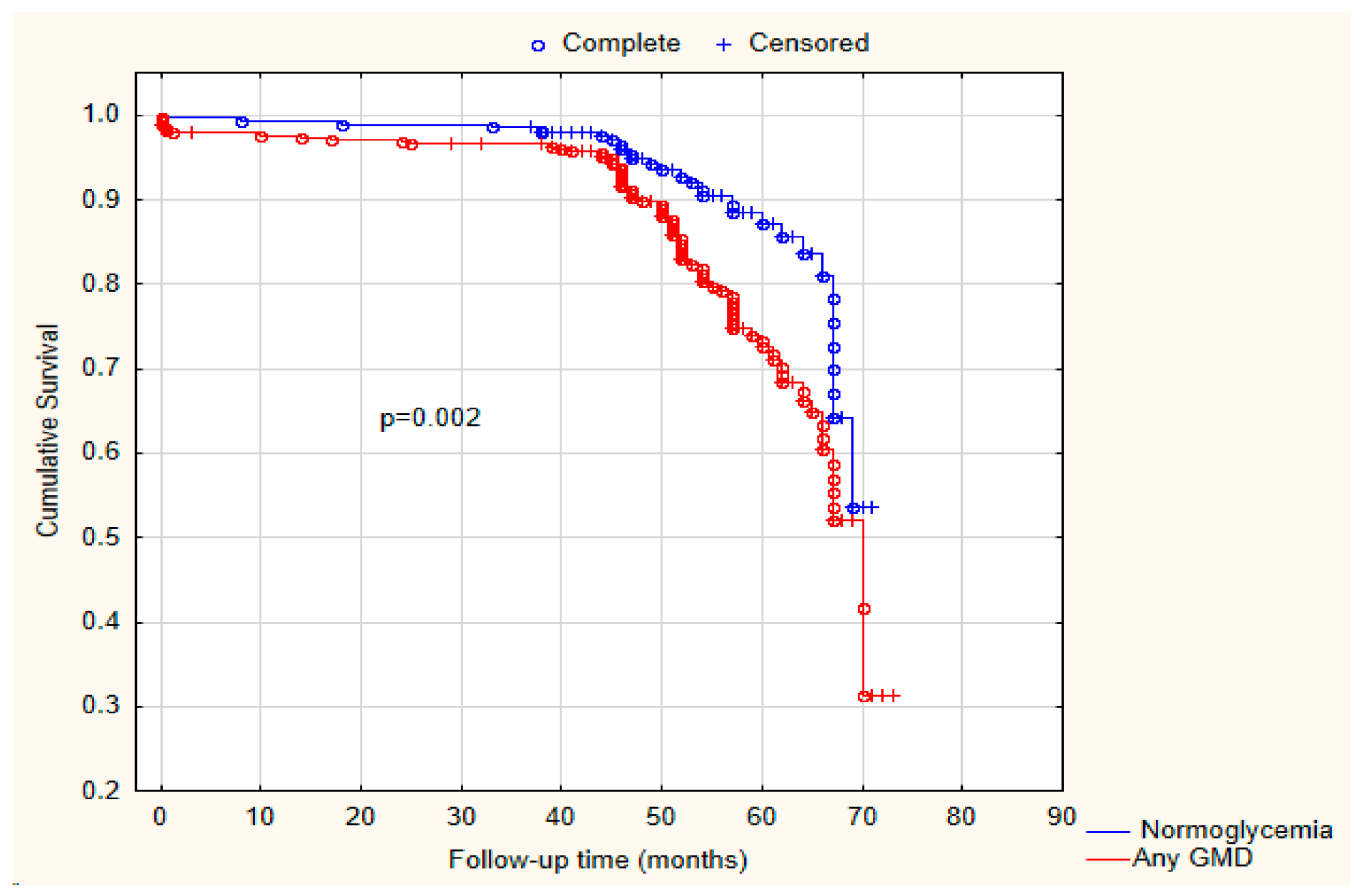
Kaplan–Meier survival curves after CABG for patients with type 2 diabetes, prediabetes, or any glucose metabolism disorder versus normoglycemic individuals. Notes: CABG—coronary artery bypass grafting; GMD—glucose metabolism disorders.

**Figure 4:**
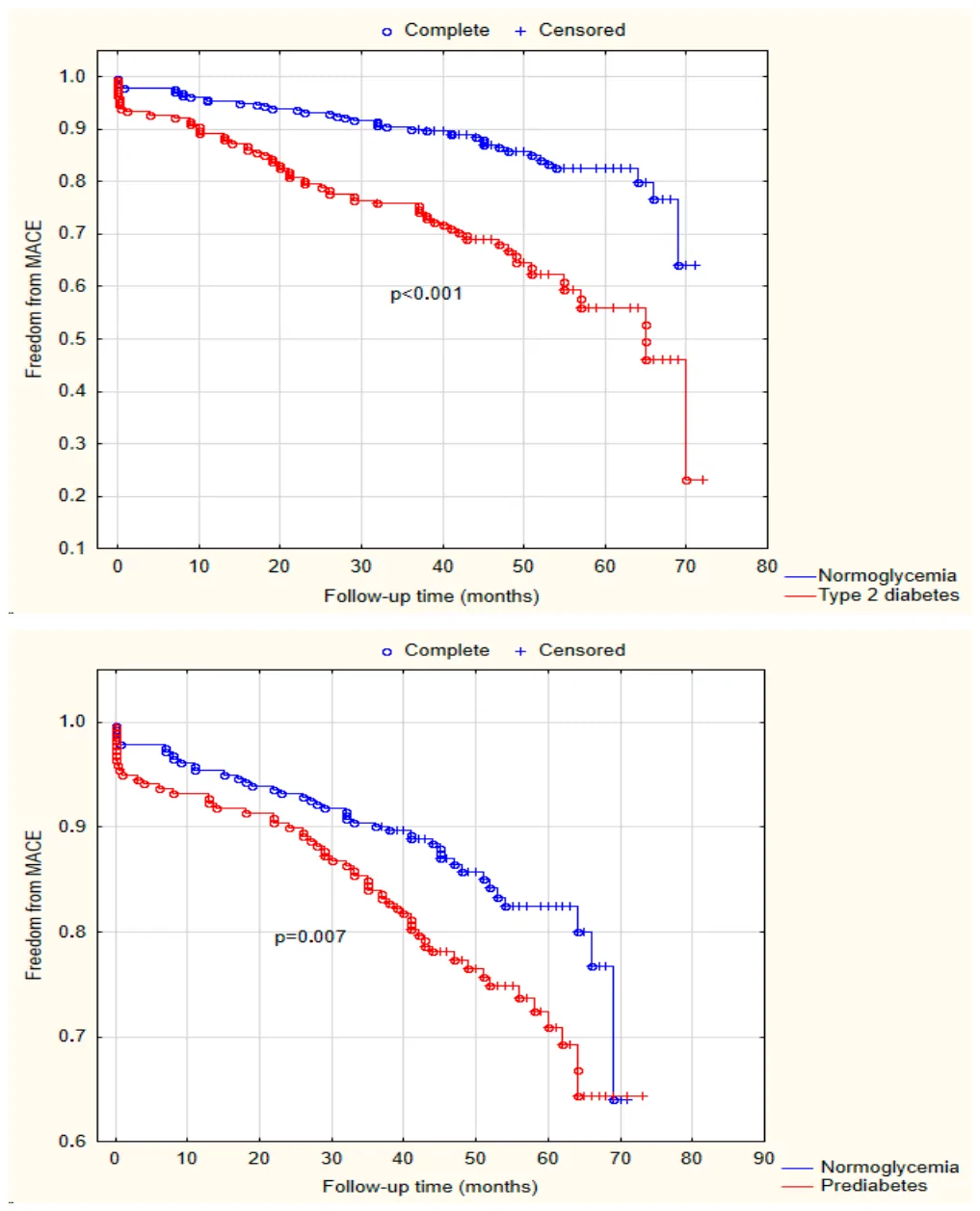

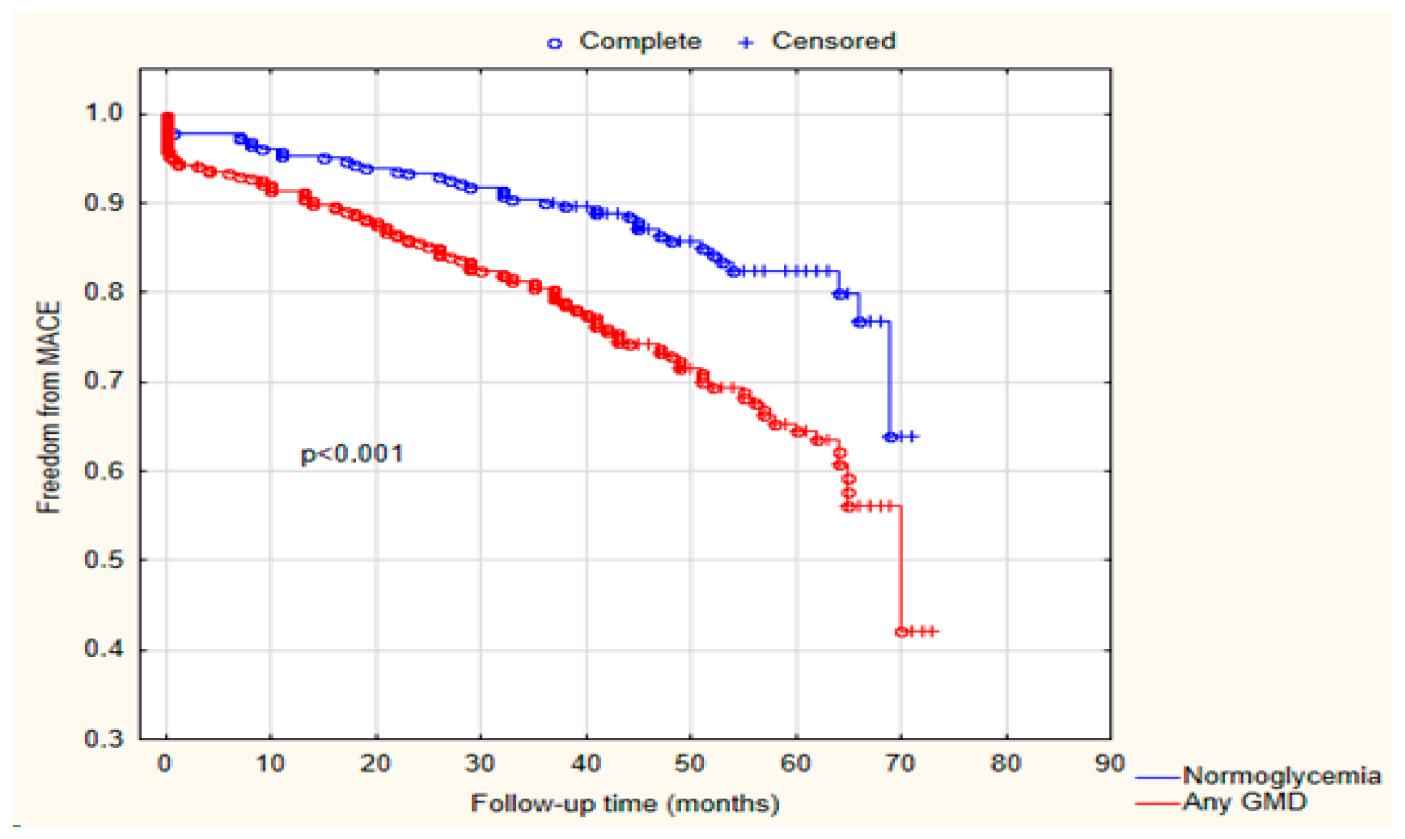
Kaplan–Meier curves for freedom from major adverse cardiovascular events after CABG for patients with type 2 diabetes, prediabetes, or any glucose metabolism disorder versus normoglycemic individuals. Notes: CABG—coronary artery bypass grafting; GMD—glucose metabolism disorders; MACE—major adverse cardiovascular events, defined as a composite of all-cause death, non-fatal myocardial infarction, or stroke.

The analysis of mid-term outcomes was complemented by the Kaplan–Meier method, which accounts for censored data and allows the cumulative probability of an outcome to be estimated as a function of follow-up time, thereby providing a clear visualization of the dynamics of the parameter under study. Actuarial survival and MACE-free survival curves were constructed over 52 months (4.2 years) after surgery (Figure 3 and Figure 4).

The Gehan–Wilcoxon test revealed better cumulative survival in patients with normoglycemia compared with those with diabetes mellitus (*p* < 0.001), but not compared with prediabetes (*p* = 0.085, Figure 3). Patients with any glucose metabolism disorder had significantly lower survival compared with the normoglycemia group (*p* = 0.002). In the normoglycemia group, the rate of freedom from MACE was significantly higher than in the other groups (*p < 0.001 compared with the groups of diabetes mellitus and all glucose metabolism disorders; p = 0.007 compared with the prediabetes group*, Figure 4). The results of the Gehan–Wilcoxon test are visually confirmed by the divergence of the actuarial curves.

**Figure 5:**
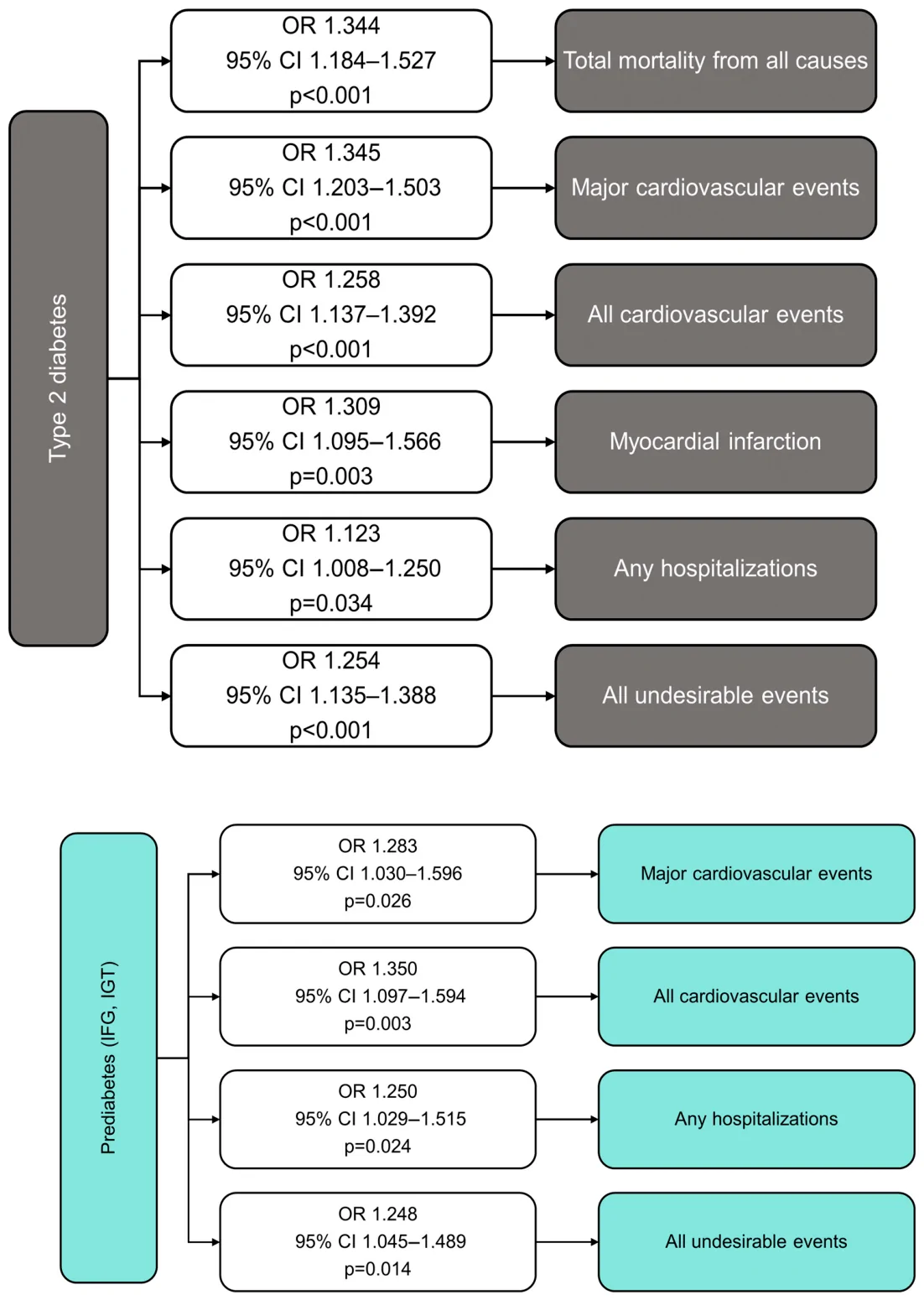

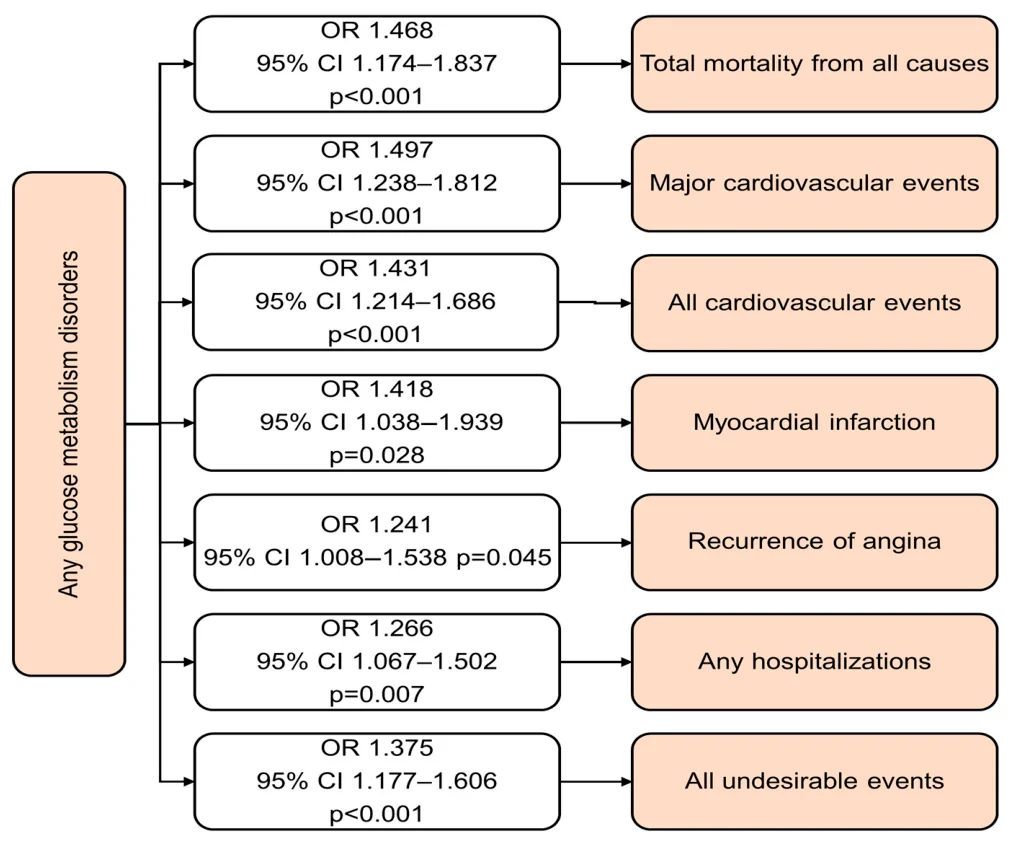
Association of type 2 diabetes, prediabetes and any glucose metabolism disorders with mid-term adverse events following coronary artery bypass grafting. Notes: OR—odds ratio; CI—confidence interval; adjusted for sex and age.

Figure 5 presents the final results of regression modeling examining the associations of type 2 diabetes, prediabetes and anyglucose metabolism disorders (GMDs) with mid-term adverse outcomes. In all figures, odds ratios, 95% CIs, and *p*-values are presented with adjustment for sex and age. Type 2 diabetes was associated with all-cause mortality (OR 1.344; *p* < 0.001), MACE (OR 1.345; *p* < 0.001), all cardiovascular events (OR 1.258; *p* < 0.001), myocardial infarction (OR 1.309; *p* = 0.003), any hospitalizations (OR 1.123; *p* = 0.034), and all adverse events (OR 1.254; *p* < 0.001). Prediabetes showed a similar but weaker pattern after adjustment for sex and age (Figure 5): MACE (OR 1.283; *p* = 0.026), all cardiovascular events (OR 1.350; *p* = 0.003), any hospitalizations (OR 1.250; *p* = 0.024), and all adverse events (OR 1.248; *p* = 0.014). Any GMD was associated with all-cause death (OR 1.468; *p* < 0.001), MACE (OR 1.497; *p* < 0.001), all cardiovascular events (OR 1.431; *p* < 0.001), myocardial infarction (OR 1.431; *p* = 0.028), angina recurrence (OR 1.241; *p* = 0.045), any hospitalizations (OR 1.266; *p* = 0.007), and all adverse events (OR 1.375; *p* < 0.001; Figure 5).

A multivariable regression analysis was then performed to assess the association of prediabetes and T2DM with the incidence of MACE (Table 6). Covariates were selected a priori based on clinical relevance and established prognostic factors; univariable analysis was used only as a preliminary screening step. Correlated variables (Pearson r > 0.7) were not entered into the same model; instead, they were tested in separate models as a sensitivity analysis. All covariates were entered simultaneously (not stepwise). Missing data were handled by complete-case analysis (listwise deletion).

In multivariable logistic regression, T2DM was associated with a 59–61% increase in the odds of MACE across different models: OR 1.59 (*p* < 0.001) after adjustment for sex, age, combined procedures, and eGFR, and OR 1.61 (*p* < 0.001) after adjustment for sex, age, total operative time, and eGFR (Table 6). The presence of any glucose metabolism disorder was also associated with MACE after adjustment for sex, age, combined procedures, and eGFR (OR 1.31; *p* < 0.001). Prediabetes remained associated with MACE after adjustment for sex, age, combined procedures, CPB duration, and eGFR (OR 1.21; *p* = 0.009). When glucose was analysed as a continuous variable, each 1 mmol/L increase was associated with a 34% increase in the odds of MACE after adjustment for sex, age, combined procedures, CPB duration, and eGFR (*p* < 0.001). In prediabetes-specific models, left atrial enlargement (OR 2.92 per 1 cm; *p* = 0.001) and higher glucose levels (OR 1.21 per 1 mmol/L; *p* = 0.028) were associated with MACE after adjustment for age and fibrinogen. Lower LV ejection fraction (*p* ≤ 0.003), older age (*p* = 0.036), and longer CPB duration (*p* ≤ 0.006) were also consistently associated with MACE (Table 6).

Subsequently, a more rigorous method, Cox proportional hazards regression, was applied; it accounts for time-to-event data (censoring), which allows for a correct assessment of the risk of outcomes over the course of the long-term postoperative period, rather than merely the occurrence of the events themselves.

An analysis of factors associated with mid-term outcomes after CABG in survival analysis using Cox proportional hazards regression is presented in Table 7 and Table 8 According to this analysis (Table 8), independent predictors of mid-term mortality were decreased renal function (*p* = 0.046), aortic cross-clamping time during CABG (*p* < 0.001), and the presence of any glucose metabolism disorder (*p* < 0.001). The same factors were independent predictors of mid-term MACE (*p* = 0.037, *p* < 0.001, and *p* < 0.001, respectively). Notably, the presence of any glucose metabolism disorder was associated with a 36.1% increase in the odds ratios of all-cause mortality and a 35.8% increase in the odds ratios of MACE, with both associations being highly significant (Wald = 12.386 and 17.584, respectively; *p* < 0.001 for both outcomes).

To verify the representativeness of the study sample, we compared patients with available long-term follow-up (*n* = 693) and those lost to follow-up (*n* = 328) using available baseline characteristics (Table 9). The comparison showed a mixed pattern: several markers of disease severity were more frequent in the followed-up group, while other markers were more frequent in the lost-to-follow-up group. Therefore, the followed-up cohort cannot be described unambiguously as either “healthier” or “sicker” than the group lost to follow-up.

On the one hand, some markers of chronic cardiovascular disease were more frequent among patients with available follow-up: III–IV CCS class angina (22.2% vs. 6.4%; *p* < 0.001), III FC CHF according to NYHA (5.0% vs. 1.2%; *p* = 0.003), and carotid artery stenosis ≥50% (29.2% vs. 17.7%; *p* < 0.001). This finding is broadly consistent with the expectation that patients with more severe chronic coronary and systemic atherosclerotic disease are more likely to reach hard endpoints and therefore to have their outcomes captured in registries and electronic health records, although this explanation remains tentative.

On the other hand, several other characteristics were more frequent in the group with unknown outcomes: unstable angina (1.7% vs. 11.9%; *p* < 0.001), intermittent claudication (3.2% vs. 7.3%; *p* = 0.003), history of PCI (9.2% vs. 14.9%; *p* = 0.006), previously diagnosed T2DM (19.2% vs. 26.8%; *p* = 0.006), and arterial hypertension (52.1% vs. 68.3%; *p* < 0.001). Preoperative glucose-lowering therapy also differed: patients with unknown outcomes were less likely to receive pre-admission insulin (2.7% vs. 22.8%; *p* < 0.001) and more likely to receive oral glucose-lowering agents (61.9% vs. 85.4%; *p* < 0.001), with sulphonylureas being the predominant class (67.3% vs. 35.7%; *p* < 0.001). No between-group differences were observed in the use of SGLT-2 inhibitors (3.5% vs. 2.9%; *p* = 0.776) or GLP-1 receptor agonists (0.9% vs. 1.2%; *p* = 0.836); prescription rates for these agents were low in both cohorts, which is explained by the study enrolment period (2016–2018).

The two groups did not differ with respect to age, BMI, obesity, the prevalence of three-vessel disease (45.9% vs. 46.6%; *p* = 0.823), left main coronary artery stenosis ≥50% (17.5% vs. 18.3%; *p* = 0.323), postoperative length of hospital stay (14.0 [12.0; 17.0] vs. 14.0 [12.0; 17.5] days; *p* = 0.209), or most background cardiovascular medications (all *p* > 0.05), with the exception of glucose-lowering therapy.

Overall, the observed differences do not point consistently in one direction. Both groups contained patients with markers of more severe disease, and the pattern of attrition is likely to reflect a combination of factors—including the differential capture of hard endpoints in patients with severe chronic coronary disease, and the differential loss of certain categories of patients (for example, those managed in other centres or presenting with acute conditions outside the planned CABG pathway). Because the net direction of the resulting selection bias cannot be predicted with certainty, the possibility of either over- or underestimation of risk should be acknowledged. This uncertainty is stated as a limitation and should be taken into account when interpreting the observed associations, although it does not preclude a meaningful analysis of the relationship between glycemic status and mid-term outcomes.

## 4. Discussion

When analyzing mid-term results after CABG surgery, the influence of preoperative GMD on the prognosis was revealed. A clear gradient in adverse event rates was evident across the three glycemic categories: normoglycemic patients experienced the fewest events, prediabetic individuals showed intermediate rates, and those with type 2 DM had the highest frequency. Kaplan–Meier survival analysis further revealed that patients with type 2 DM had significantly worse long-term survival than their normoglycemic counterparts. The presence of prediabetes did not increase mortality during the follow-up period, but was associated with an increased incidence of MACE.

The adverse impact of type 2 DM on prognosis after CABG is undisputed [6,17]. Results of a nationwide study based on data from the Dutch Cardiology Registry showed that DM is associated with increased mortality after various cardiac surgeries [6]. After adjustment for baseline characteristics, DM remained associated with higher mortality at 3 months (OR 1.35; *p* < 0.001) and 1 year after CABG (OR 1.43; *p* < 0.001) [6]. Therefore, the attention of researchers has mainly been focused on a comparative study of the effectiveness of various myocardial revascularization methods in patients with DM [3,18]. For example, a meta-analysis by Zhai C. et al., which included 16 studies (18,224 patients), demonstrated the advantages of CABG over PCI in patients with DM and complex CAD [3]. PCI was associated with an increased risk of MACE (OR 1.59; 95% CI 1.38–1.85), cardiac death (OR 1.76; 95% CI 1.11–2.80), MI (OR 1.98; 95% CI 1.53–2.57), and repeat revascularization (OR 2.61; 95% CI 2.08–3.29). Moreover, in the long-term period, a significant increase in the risk of overall mortality was observed after PCI (OR 1.32; 95% CI 1.04–1.67), while CABG was associated with a higher incidence of strokes in the medium term (OR 0.39; 95% CI 0.23–0.66) [3].

At the same time, studies of the impact of prediabetes on the mid-term and long-term outcomes of CABG have yielded conflicting results. Separately, our own observations indicated that the prevalence of any GMD (encompassing both DM and prediabetes) increased significantly from 41.1% to 52.2% at one year following CABG (*p* < 0.001) [19], suggesting that prediabetic patients may face a deteriorating clinical outlook. A retrospective cohort study [20] analyzed 3741 patients, including groups with DM, prediabetes, and normoglycemia. Although the overall 1-year survival did not differ between the groups, a trend toward an increased incidence of MACE in patients with DM (96.8%) compared with prediabetes (98.1%) and normoglycemia (97.4%) suggests a potential negative impact of hyperglycemia during longer follow-up. In a cohort of 497 patients who underwent primary CABG in Stockholm, Sweden, between 2005 and 2013, the association between preoperative glucose metabolism disorders and 10-year survival was analyzed [9]. All patients without previously diagnosed DM underwent OGTT preoperatively, as in our case. A key finding of the study was the absence of statistically significant differences in long-term survival between the groups according to multivariate analysis. Cumulative 10-year survival was 77% in the normoglycemic group, 83% in the prediabetes group, and 71% in patients with newly diagnosed DM; these differences did not reach statistical significance after adjusting for confounding factors [9]. It is possible that direct myocardial revascularization and subsequent follow-up may offset the potentially negative impact of GMD on long-term prognosis.

Findings from another study involving CAD patients with a prior history of MI and/or coronary revascularization, who were followed prospectively for 5 years, showed that both manifest diabetes mellitus and prediabetes (i.e., impaired fasting glucose) independently increased the risk of death from any cause when compared with normoglycemia. The adjusted odds ratios were 1.63 (*p* = 0.043) for DM and 2.25 (*p* < 0.0001) for prediabetes [21]. Moreover, impaired fasting glucose was strongly associated with five-year cardiovascular mortality (OR 3.84; 95% CI 2.19–6.73), corresponding to a nearly 4-fold increase in the likelihood of cardiovascular death, whereas DM was associated with a 2-fold increase (OR 1.96; 95% CI 1.06–3.63) [21].

During long-term postoperative follow-up of patients, no effect of prediabetes or newly diagnosed DM before CABG on long-term survival was found compared with normoglycemic patients [9]. Similar results were obtained by Fallahzadeh et al. [10]: prediabetes showed no prognostic impact on overall survival or cardiovascular event-free survival after CABG. However, patients with prediabetes after elective percutaneous coronary intervention (PCI) had a higher mortality rate and a trend toward a higher rate of restenosis over 2 years compared with normoglycemic patients [8]. The data from a later meta-analysis were also compelling: among patients undergoing PCI for CAD, the risk of all-cause and cardiovascular mortality, major adverse cardiovascular events (MACE), and myocardial infarction (MI) was higher in patients with prediabetes compared with normoglycemic patients, but lower compared with patients with DM [11].

The results of our study indicate that the identified GMD have a significant negative impact on the mid-term outcomes of CABG. Patients with any GMD had significantly lower cumulative survival and freedom from MACE compared to the normoglycemic group. DM and prediabetes diagnosed before CABG were independent factors associated with all-cause mortality and cardiovascular events during four-year follow-up after surgery.

### Study Limitations

This study has several limitations that should be considered when interpreting the findings. First, although 1021 patients with known glycemic status formed the original cohort, follow-up information sufficient for analysis was available for only 693 patients (67.9%), and a further 349 patients were excluded at the screening stage because their glycemic status was unknown. We compared included and excluded patients using available baseline characteristics (Table 9) and found a bidirectional pattern of attrition bias: patients with severe cardiovascular disease were more likely to be retained because hard endpoints were completely captured, whereas patients with milder diabetes and hypertension who did not reach endpoints were more likely to be lost to follow-up. Although this bias is likely to shift effect estimates towards an overestimation of risk rather than to invalidate the observed associations, selection and attrition bias cannot be fully excluded.

Second, this was an observational study, and residual confounding cannot be excluded despite adjustment for clinically relevant covariates. The three groups differed substantially in age, sex, body mass index, duration of hypertension and coronary artery disease, EuroSCORE II, and several operative characteristics. Although we used multivariable logistic regression and Cox proportional hazards models with a priori selected covariates, and avoided entering correlated variables into the same model, unmeasured confounders (e.g., frailty, socioeconomic status, adherence to therapy, and detailed diabetes duration/treatment intensity) may still have influenced the results.

Third, outcomes were ascertained from the institutional electronic medical record system, civil registry data, structured telephone interviews, and available medical documentation, but they were not independently adjudicated by a blinded endpoint committee, and the investigators assessing outcomes were not blinded to glycemic status. Although events reported by patients or relatives were verified against medical records whenever possible, some misclassification or under-ascertainment of events cannot be excluded. In addition, MACE was analysed as the time to the first qualifying event; recurrent events were not included in the primary MACE analysis, which may underestimate the total disease burden.

Fourth, CABG was performed between 2016 and 2018. During that period, GLP-1 receptor agonists and SGLT-2 inhibitors were used in only 1.2% and 2.9% of patients with type 2 diabetes, respectively. This differs substantially from contemporary diabetes and cardiovascular practice and limits the generalizability of our findings to current cohorts. Treatment strategies for diabetes, heart failure, and atherosclerotic cardiovascular disease have changed considerably since the enrolment period, and the prognostic impact of glucose metabolism disorders may be modified by contemporary therapies.

Fifth, prediabetes was defined according to WHO glucose criteria (impaired fasting glucose and/or impaired glucose tolerance), consistent with the clinical guidelines adopted in the Russian Federation and endorsed by the European Association for the Study of Diabetes; HbA1c was not used as a standalone criterion. We acknowledge that alternative diagnostic criteria exist, particularly the American Diabetes Association HbA1c threshold, which would reclassify some patients and might influence the observed associations. Similarly, patients with previously diagnosed and newly detected type 2 diabetes were analysed together in the main models; although we report their separate distribution in Table 8, combining these groups may obscure differences in disease duration and treatment intensity. Prediabetes was defined using WHO glucose criteria; HbA1c was not used as a standalone criterion. Alternative definitions, such as the ADA HbA1c threshold, would reclassify some patients.

Sixth, the study evaluated the prognosis associated with baseline glycemic status; it was not designed to demonstrate that screening for or treating prediabetes improves outcomes after CABG. The clinical implications of our findings should therefore remain proportionate to this observational question. Finally, the sample size was modest for some subgroup and category-specific analyses (e.g., mortality in prediabetes), and the absence of a statistically significant association in these analyses should not be interpreted as evidence that an effect is absent.

It should be noted that, for the Cox regression analysis, we had to select “any glucose metabolism disorder” as a predictor, as there were an insufficient number of events to consider prediabetes and established diabetes separately. Since combining prediabetes and established diabetes in a Cox regression analysis may obscure clinically important differences between these conditions, this also represents one of the study’s limitations.

## 5. Conclusions

An analysis of mid-term outcomes after CABG revealed an impact of preoperative GMD. For most adverse events, an increasing frequency was observed, from the lowest in the normoglycemic group to the higher in the prediabetes group and the highest in the DM group. Long-term mortality percentages were worse in patients with DM compared to the normoglycemic group (29.2% vs. 11.2%, *p* < 0.001). The presence of prediabetes did not increase mortality during the follow-up period (15.7% vs. 11.2), but was associated with an increased incidence of MACE (24.6% vs. 15.85%, *p* = 0.012). According to this analysis Cox proportional hazards regression, independent predictors of mid-term mortality were decreased renal function (*p* = 0.046), aortic cross-clamping time during CABG (*p* < 0.001), and the presence of glucosemetabolism disorders (*p* < 0.001). The same factors were independent predictors of mid-term MACE (*p* = 0.037, *p* < 0.001, and *p* < 0.001, respectively). The results of this study confirm that DM and prediabetes are associated with adverse events during a 4-year follow-up period after CABG, in a sample with an active diagnostic approach to detecting GMD.

## Figures and Tables

**Table 1:** Adverse events during follow-up according to glucose metabolism status (*n* = 693).

Characteristic, *n* (%)	Group 1 Normoglycemia (*n* = 293)	Group 2 Prediabetes (*n* = 229)	Group 3 Type 2 Diabetes (*n* = 171)	*p*-Value
All adverse events	125 (42.6)	125 (54.4)	114 (66.5)	<0.001 <0.001 ^1-3^ 0.007 ^1-2^ 0.016 ^2-3^
Major adverse cardiovascular events (MACE)	46 (15.7)	56 (24.5)	67 (39.2)	<0.001 <0.001 ^1-3^ 0.002 ^1-2^
All cardiovascular events	79 (27.1)	91 (39.9)	84 (49.1)	<0.001 <0.001 ^1-3^ 0.002 ^1-2^
All-cause mortality	31 (10.6)	35 (15.3)	49 (28.7)	<0.001 <0.001 ^1-3^ 0.001 ^2-3^
Cardiovascular mortality	17 (5.8)	21 (9.1)	30 (17.5)	<0.001 <0.001 ^1-3^ 0.012 ^2-3^
Myocardial infarction	15 (5.2)	17 (7.5)	22 (12.7)	0.011 0.005 ^1-3^
Stroke	10 (3.5)	12 (5.4)	13 (7.6)	0.154
Percutaneous coronary intervention	16 (5.3)	8 (3.6)	12 (7.0)	0.313
Carotid artery intervention	8 (2.8)	9 (3.9)	3 (1.8)	0.240
Lower extremity artery intervention	5 (1.8)	6 (2.7)	4 (2.3)	0.780
Recurrent angina	40 (13.7)	45 (19.7)	31 (18.0)	0.142
Emergency hospitalizations for CVD	63 (21.6)	62 (27.1)	53 (30.8)	0.079

Notes: Data are presented as *n* (%). For overall three-group comparisons, the global *p*-value was calculated; when significant differences emerged (*p* < 0.05), pairwise comparisons were performed with a Bonferroni correction (critical significance threshold set at 0.017); *p*—result of Kruskal-Wallis test, *p* ^1-2, 2-3, 1-3^—*p* in pairwise comparison of groups 1-2, 2-3, 1-3. Only statistically significant pairwise *p*-values (*p* < 0.017) are displayed. MACE was defined as a composite of all-cause death, non-fatal myocardial infarction, or stroke. CVD—cardiovascular disease.

**Table 2:** Baseline clinical characteristics of patients according to glycemic status.

Characteristic	Group 1 Normoglycemia (*n* = 293)	Group 2 Prediabetes (*n* = 229)	Group 3 Type 2 Diabetes (*n* = 171)	*p*-Value
Male, *n* (%)	244 (83.3)	187 (81.7)	116 (67.8)	<0.001 <0.001 ^1-3^
Female, *n* (%)	49 (16.7)	42 (18.3)	55 (32.2)	<0.001 <0.001 ^1-3^
Age (years), Me (Q1–Q3)	58.8 [56.5; 64.7]	64.0 [62.1; 67.7]	69.4 [68.0; 69.4]	0.005 0.006 ^1-3^
BMI (kg/m^2^), Me (Q1–Q3)	25.0 [24.2; 27.7]	27.7 [26.9; 30.5]	28.7 [29.4; 31.2]	<0.001 <0.001 ^1-2,1-3^
Obesity, *n* (%)	61 (20.9)	65 (28.6)	58 (34.2)	0.024
Arterial hypertension, *n* (%)	143 (48.8)	135 (58.9)	94 (55.0)	0.301
Hypertension duration (years), Me (Q1–Q3)	4.0 [3.0; 5.0]	10.0 [8.0; 10.0]	15.0 [10.0; 17.5]	<0.001 <0.001 ^1-2,1-3^
CAD duration (years), Me (Q1–Q3)	1.0 [1.0; 2.0]	2.0 [2.0; 5.0]	6.0 [2.0; 9.0]	0.004 0.003 ^1-3^ 0.042 ^2-3^
CHF, NYHA class I, *n* (%)	5 (1.8)	10 (4.3)	7 (3.9)	0.231
CHF, NYHA class II, *n* (%)	275 (93.9)	204 (89.1)	157 (91.8)	0.086
CHF, NYHA class III, *n* (%)	13 (4.3)	15 (6.5)	7 (3.9)	0.412
Angina class III–IV, *n* (%)	49 (16.7)	34 (14.8)	34 (19.9)	0.107
Unstable angina, *n* (%)	5 (1.7)	4 (1.7)	3 (1.8)	0.999
Smoking, *n* (%)	45 (15.3)	43 (18.6)	19 (11.0)	0.103
Heart rhythm disturbances, *n* (%)	68 (23.2)	71 (31.0)	45 (26.3)	0.119
History of myocardial infarction, *n* (%)	104 (35.4)	84 (36.7)	66 (38.5)	0.794
History of stroke, *n* (%)	13 (4.4)	6 (2.6)	9 (5.3)	0.373
History of carotid endarterectomy, *n* (%)	1 (0.3)	0 (0)	0 (0)	0.504
History of pacemaker implantation, *n* (%)	0 (0)	2 (0.9)	1 (0.6)	0.301
History of PCI, *n* (%)	27 (9.2)	22 (9.6)	15 (8.8)	0.959
History of CABG, *n* (%)	2 (0.7)	0 (0)	0 (0)	0.254
Dyslipidemia, *n* (%)	78 (26.6)	73 (31.9)	55 (32.2)	0.298
Peripheral artery disease, *n* (%)	23 (7.9)	21 (9.2)	24 (13.8)	0.122
Intermittent claudication, *n* (%)	10 (3.4)	6 (2.6)	6 (3.5)	0.838
History of amputation, *n* (%)	2 (0.7)	0 (0)	1 (0.6)	0.468
Prior peripheral arterial surgery, *n* (%)	3 (1.0)	2 (0.9)	0 (0)	0.436
Thyroid disease, *n* (%)	4 (1.4)	3 (1.3)	2 (1.2)	0.985
Chronic kidney disease, *n* (%)	23 (7.8)	19 (8.5)	18 (10.5)	0.625
EuroSCORE II (%), Me (Q1–Q3)	0.9 [0.9; 1.5]	1.5 [1.3; 1.9]	1.9 [1.9; 2.7]	0.002 0.003 ^1-3^ 0.020 ^2-3^

Notes: Me (Q1–Q3)—median with first and third quartiles. For overall three-group comparisons, the global *p*-value was calculated; when significant differences emerged (*p* < 0.05), pairwise comparisons were performed with a Bonferroni correction (critical significance threshold set at 0.017); *p*—result of Kruskal-Wallis test, *p* ^1-2, 2-3, 1-3—^*p* in pairwise comparison of groups 1-2, 2-3, 1-3. BMI—body mass index; CAD—coronary artery disease; CHF—chronic heart failure; NYHA—New York Heart Association; PCI—percutaneous coronary intervention.

**Table 3:** Intraoperative and perioperative characteristics of coronary artery bypass grafting (*n* = 693).

Characteristic	Group 1 Normoglycemia (*n* = 293)	Group 2 Prediabetes (*n* = 229)	Group 3 Type 2 Diabetes (*n* = 171)	*p*-Value
Elective CABG, *n* (%)	258 (88.1)	203 (88.6)	152 (89.0)	0.950
Priority elective CABG, *n* (%)	35 (11.9)	26 (11.4)	19 (11.1)	0.950
On-pump surgery, *n* (%) 591	246 (84.0)	202 (88.2)	143 (83.6)	0.398
Off-pump surgery, *n* (%)	47 (16.0)	27 (11.8)	28 (16.3)	0.398
Isolated CABG, *n* (%)	277 (94.5)	209 (91.3)	160 (93.6)	0.357
Combined procedures, *n* (%)	16 (5.5)	20 (8.7)	11 (6.4)	0.357
• Carotid endarterectomy, *n* (%)	2 (0.7)	2 (0.9)	1 (0.6)	0.931
• Ventriculoplasty, *n* (%)	8 (2.7)	11 (4.8)	7 (4.1)	0.428
• Radiofrequency ablation, *n* (%)	0 (0)	1 (0.4)	1 (0.6)	0.451
• Mitral valve surgery, *n* (%)	2 (0.7)	1 (0.4)	1 (0.6)	0.929
• Aortic valve surgery, *n* (%)	1 (0.3)	2 (0.9)	0 (0)	0.406
Cardiopulmonary bypass time (min), Me (Q1–Q3)	85.0 [72.0; 106.0]	81.0 [67.0; 100.0]	83.0 [68.0; 114.0]	0.665
Cardioplegia deliveries (*n*), Me (Q1–Q3)	2.0 [2.0; 3.0]	2.0 [2.0; 3.0]	2.0 [2.0; 3.0]	0.912
Aortic crossclamp time (min), Me (Q1–Q3)	55.0 [45.0; 71.0]	54.0 [44.0; 68.0]	54.5 [44.0; 74.0]	0.838
Number of grafts, Me (Q1–Q3)	2.0 [2.0; 3.0]	3.0 [2.0; 3.0]	3.0 [2.0; 3.0]	0.057
Minimum body temperature (°C), Me (Q1–Q3)	35.6 [35.3; 35.7]	35.5 [35.3; 35.7]	35.6 [35.3; 35.7]	0.883
Total operative time (h), Me (Q1–Q3)	3.4 [3.2; 4.2]	3.5 [3.2; 4.3]	3.4 [3.1; 4.4]	0.751
Mechanical ventilation duration (h), Me (Q1–Q3)	6.0 [5.0; 7.5]	8.0 [6.1; 13.0]	7.1 [5.0; 9.4]	0.002 0.001 ^1-2^
ICU stay (h), Me (Q1–Q3)	21.0 [21.0; 22.0]	22.0 [22.0; 43.0]	21.0 [19.7; 22.6]	0.106
ICU stay (days), Me (Q1–Q3)	0.9 [0.9; 1.0]	0.9 [0.9; 1.8]	0.9 [0.8; 0.9]	0.147
Preoperative medications, *n* (%)				
• Acetylsalicylic acid	261 (89.1)	214 (93.4)	151 (88.3)	0.145
• ACE inhibitors	203 (69.3)	162 (70.7)	129 (75.4)	0.284
• Angiotensin II receptor antagonists	90 (30.7)	66 (28.8)	41 (24.0)	0.272
• Beta-blockers	257 (87.7)	202 (88.2)	155 (90.6)	0.643
• Calcium channel blockers	186 (63.5)	134 (58.5)	116 (67.8)	0.137
• Loop diuretics	100 (34.1)	71 (31.0)	63 (36.8)	0.462
• Thiazide diuretics	129 (44.0)	93 (40.6)	85 (49.7)	0.152
• Statins	268 (91.5)	195 (85.2)	153 (89.5)	0.047
Perioperative antidiabetic therapy (only for patients with type 2 DM)				
• Oral glucose-lowering agents, *n* (%)	-	-	146 (85.4)	-
• Sulfonylureas, *n* (%)	-	-	61 (35.7)	-
• Metformin, *n* (%)	-	-	82 (48.0)	-
• DPP-4 inhibitors, *n* (%)	-	-	7 (4.1)	-
• GLP-1 receptor agonists, *n* (%)	-	-	2 (1.2)	-
• SGLT-2 inhibitors, *n* (%)	-	-	5 (2.9)	-
• Pre-hospital insulin, *n* (%)	-	-	39 (22.8)	-
• In-hospital insulin, *n* (%)	-	-	107 (62.6)	-
Total daily insulin dose before CABG, IU/day, Me [LQ; UQ]	-	-	28.0 [21.0; 38.0]	-
Total insulin dose within first 24 h after CABG, IU/day, Me [LQ; UQ]	-	-	13.0 [0.0; 29.0]	-

Notes: For overall three-group comparisons, the global *p* value was calculated; when significant differences emerged (*p* < 0.05), pairwise comparisons were performed with a Bonferroni correction (critical significance threshold set at 0.017); *p*—result of Kruskal-Wallis test, *p* ^1-2^—*p* in pairwise comparison of groups 1-2. CABG—coronary artery bypass grafting; ACE—angiotensin converting enzyme; ICU—intensive care unit; CABG—coronary artery bypass grafting; DPP-4—dipeptidyl peptidase-4; GLP-1—glucagon-like peptide-1; SGLT-2—sodium–glucose cotransporter-2.

**Table 4:** Preoperative cardiac and vascular instrumental findings according to glycemic status.

Parameter	Group 1 Normoglycemia (*n* = 293)	Group 2 Prediabetes (*n* = 229)	Group 3 Type 2 Diabetes (*n* = 171)	*p*-Value
Carotid artery stenosis ≥ 50% pre-CABG	76 (25.9)	73 (31.8)	53 (31.0)	0.616
Echocardiography, Me (Q1-Q3)				
LV end-diastolic dimension (cm)	5.6 [5.3; 6.1]	5.7 [5.3; 6.2]	5.6 [5.2; 6.2]	0.770
LV end-systolic dimension (cm)	3.6 [3.4; 4.3]	3.7 [3.4; 4.4]	3.7 [3.3; 4.4]	0.713
Left atrial diameter (cm)	4.3 [3.9; 4.6]	4.3 [4.0; 4.7]	4.3 [4.0; 4.7]	0.042
Interventricular septal (cm)	1.1 [1.0; 1.2]	1.1 [1.0; 1.2]	1.1 [1.0; 1.3]	0.089
Aortic root diameter (cm)	3.6 [3.4; 3.8]	3.6 [3.3; 3.8]	3.5 [3.3; 3.8]	0.881
LV ejection fraction (%)	63.0 [53.0; 67.0]	62.0 [53.5; 65.0]	63.0 [53.0; 67.0]	0.682
LV mass (Devereux & Reichek, g)	293.8 [250.4; 366.7]	312.2 [258.8; 391.1]	320.6 [267.2; 386.8]	0.192
Coronary angiography data, *n* (%)				
One-vessel disease *	51 (17.4)	46 (20.1)	12 (7.0)	<0.001 <0.001 ^2-3^
Two-vessel disease *	114 (38.6)	52 (22.7)	73 (42.7)	<0.001 <0.001 ^1-2, 2-3^
Three-vessel disease *	119 (40.6)	122 (53.7)	77 (45.0)	0.014 <0.001 ^1-2^ 0.029 ^2-3^
Left main stem stenosis ≥ 50%	54 (18.4)	41 (17.9)	26 (15.2)	0.139

Notes: For overall three-group comparisons, the global *p*-value was calculated; when significant differences emerged (*p* < 0.05), pairwise comparisons were performed with a Bonferroni correction (critical significance threshold set at 0.017); *p*—result of Kruskal-Wallis test, *p* ^1-2, 2-3, 1-3^—*p* in pairwise comparison of groups 1-2, 2-3, 1-3. LV—left ventricle; CABG—coronary artery bypass grafting. * number of major coronary arteries with stenosis ≥70%.

**Table 5:** Preoperative laboratory values by glycemic status group.

Parameter, Me (Q1–Q3)	Group 1 Normoglycemia (*n* = 293)	Group 2 Prediabetes (*n* = 229)	Group 3 Type 2 Diabetes (*n* = 171)	*p*-Value
Fasting glucose, mmol/L	5.2 [4.9; 5.6]	6.0 [5.3; 6.3]	7.3 [6.1; 8.8]	<0.001 <0.001 ^1-2, 2-3, 1-3^
HbA1c, %	5.2 [5.0; 5.5]	5.7 [5.3; 6.0]	6.8 [6.1; 7.9]	<0.001 <0.001 ^1-2, 2-3, 1-3^
Total cholesterol, mmol/L	4.6 [3.8; 5.5]	4.6 [3.9; 5.4]	5.2 [3.9; 6.2]	0.657
HDL cholesterol, mmol/L	1.1 [0.9; 1.3]	1.1 [0.9; 1.4]	1.0 [0.8; 1.3]	0.030
LDL cholesterol, mmol/L	2.7 [2.1; 3.5]	2.7 [2.2; 3.5]	2.9 [2.2; 4.1]	0.991
Triglycerides, mmol/L	1.4 [1.1; 2.0]	1.5 [1.3; 2.1]	1.7 [1.3; 2.4]	0.001 <0.001 ^1-3^
Creatinine, µmol/L	78 [78; 89]	76 [67; 91]	81 [69; 94]	0.835
Urea, mmol/L	5.8 [4.5; 6.9]	5.6 [4.6; 6.2]	6.1 [5.1; 7.2]	0.200
eGFR (CKD-EPI), mL/min/1.73 m^2^	92.9 [78.2; 100.8]	95.7 [80.9; 102.5]	89.5 [75.1; 99.0]	0.316
aPTT, s	28.0 [26.0; 29.8]	29.0 [26.7; 33.4]	26.8 [26.0; 29.5]	0.053
Fibrinogen, g/L	3.8 [3.5; 4.5]	4.2 [3.5; 4.7]	3.9 [3.5; 4.8]	0.078
SFMC, mg/100 mL	4.0 [3.5; 6.5]	5.0 [4.0; 7.5]	4.0 [3.5; 7.5]	0.122

Notes: For overall three-group comparisons, the global *p*-value was calculated; when significant differences emerged (*p* < 0.05), pairwise comparisons were performed with a Bonferroni correction (critical significance threshold set at 0.017); *p*—result of Kruskal-Wallis test, *p* ^1-2, 2-3, 1-3^—*p* in pairwise comparison of groups 1-2, 2-3, 1-3. CKD-EPI—Chronic Kidney Disease Epidemiology Collaboration; eGFR—estimated glomerular filtration rate; HbA1c—glycated hemoglobin; HDL—high-density lipoprotein; LDL—low-density lipoprotein; aPTT—activated partial thromboplastin time; SFMC—soluble fibrin monomer complexes.

**Table 6:** Multivariable logistic regression models for major adverse cardiovascular events.

Predictor	OR (95% CI)	*p*-Value
Model 1. T2DM; *p* < 0.001; adjusted for sex, age, combined procedures, eGFR CKD EPI.		
Type 2 diabetes (vs. normoglycemia)	1.59 (1.33–1.90)	<0.001
LV ejection fraction (per 1%)	0.94 (0.91–0.97)	<0.001
Model 2. Prediabetes; *p* < 0.001; adjusted for sex, age, combined procedures, CPB duration, eGFR CKD EPI.		
Prediabetes (vs. normoglycemia)	1.21 (1.05–1.40)	0.009
LV ejection fraction (per 1%)	0.95 (0.92–0.98)	0.003
Model 3. Any GMD; *p* < 0.001; adjusted for sex, age, combined procedures, eGFR CKD EPI.		
Any GMD (vs. normoglycemia)	1.31 (1.16–1.48)	<0.001
LV ejection fraction (per 1%)	0.95 (0.92–0.97)	<0.001
Model 4. Glucose as a continuous variable; *p* = 0.001; adjusted for age and fibrinogen.		
Glucose (per 1 mmol/L)	1.21 (1.02–1.44)	0.028
Left atrial dimension (per 1 cm)	2.92 (1.50–5.66)	0.001
Model 5. Glucose as continuous variable; *p* < 0.001; adjusted for sex, age, combined procedures, CPB duration, eGFR CKD EPI.		
Glucose (per 1 mmol/L)	1.34 (1.14–1.56)	<0.001
LV ejection fraction (per 1%)	0.95 (0.93–0.98)	<0.001
CPB duration (per 5 min)	1.06 (1.02–1.11)	0.006
Model 6. T2DM; *p* < 0.001; adjusted for sex, age, combined procedures, eGFR CKD EPI.		
Type 2 diabetes (vs. normoglycemia)	1.55 (1.31–1.83)	<0.001
LV end-diastolic dimension (per 1 cm)	1.98 (1.20–3.27)	0.007
Model 7. T2DM; *p* < 0.001; adjusted for sex, age, combined procedures, CPB duration.		
Type 2 diabetes (vs. normoglycemia)	1.32 (1.16–1.51)	<0.001
Age (per 1 year)	1.03 (1.00–1.07)	0.036
LV ejection fraction (per 1%)	0.96 (0.94–0.98)	<0.001
CPB duration (per 5 min)	1.05 (1.01–1.08)	0.006

Notes: Only statistically significant predictors (*p* < 0.05) are shown. Covariates listed in the model header were included but were not statistically significant. OR—odds ratio; CI—confidence interval; LV—left ventricular; CPB—cardiopulmonary bypass; eGFR CKD EPI—estimated glomerular filtration rate; GMD—glucose metabolism disorder. In Models 1, 6 and 7, prediabetes patients were excluded. In Model 3, any GMD includes both prediabetes and T2DM. In Model 2, T2DM patients were excluded. Models 4 and 5 analyze glucose as a continuous variable.

**Table 7:** Predictors of mid-term all-cause mortality after CABG in survival analysis using Cox proportional hazards regression.

	Enter Method						95.0% CI for Exp(B)	
	B	SE	Wald	df	Sig.	Exp(B)	Lower	Upper
Sex	−0.187	0.349	0.286	1	0.593	0.830	0.418	1.646
Age	0.003	0.020	0.025	1	0.874	1.003	0.965	1.043
EuroSCORE II	0.025	0.037	0.473	1	0.491	1.026	0.954	1.102
GFR by CKD EPI	−0.014	0.008	3.265	1	0.071	0.986	0.971	1.001
Aortic cross-clamp time	0.014	0.006	6.788	1	0.009	1.015	1.004	1.026
Number of shunts	−0.040	0.202	0.040	1	0.842	0.960	0.646	1.427
Combined operations	0.091	0.362	0.063	1	0.802	1.095	0.539	2.226
Total operative time	−0.078	0.172	0.205	1	0.651	0.925	0.661	1.295
Any glucose metabolism disorder	0.314	0.089	12.350	1	0.000	1.368	1.149	1.630
Forward LR method								
CFR by CKD EPI	−0.014	0.007	3.979	1	0.046	0.986	0.973	1.000
Aortic cross-clamp time	0.014	0.003	15.565	1	0.000	1.014	1.007	1.020
Any glucose metabolism disorder	0.308	0.088	12.386	1	0.000	1.361	1.146	1.615

Notes: CABG—coronary artery bypass grafting, GFR—glomerular filtration rate, CKD-EPI—Chronic Kidney Disease Epidemiology Collaboration.

**Table 8:** Predictors of mid-term MACE after CABG in survival analysis using Cox proportional hazards regression.

	Enter Method						95.0% CI for Exp(B)	
	B	SE	Wald	df	Sig.	Exp(B)	Lower	Upper
Sex	0.111	0.284	0.154	1	0.695	1.118	0.641	1.951
Age	−0.003	0.017	0.039	1	0.843	0.997	0.964	1.030
EuroSCORE II	−0.009	0.055	0.025	1	0.875	0.991	0.891	1.104
GFR by CKD EPI	−0.012	0.007	3.155	1	0.076	0.988	0.975	1.001
Aortic cross-clamp time	0.012	0.005	6.953	1	0.008	1.012	1.003	1.022
Number of shunts	0.086	0.173	0.247	1	0.619	1.090	0.777	1.529
Combined operations	0.152	0.310	0.240	1	0.624	1.164	0.634	2.139
Total operative time	−0.065	0.143	0.204	1	0.651	0.937	0.708	1.241
Any glucose metabolism disorder	0.306	0.075	16.814	1	0.000	1.358	1.173	1.572
Forward LR method								
CFR by CKD EPI	−0.013	0.006	4.351	1	0.037	0.987	0.976	0.999
Aortic cross-clamp time	0.011	0.003	14.145	1	0.000	1.011	1.005	1.018
Any glucose metabolism disorder	0.308	0.073	17.584	1	0.000	1.360	1.178	1.571

Notes: CABG—coronary artery bypass grafting, GFR—glomerular filtration rate, CKD-EPI—Chronic Kidney Disease Epidemiology Collaboration.

**Table 9:** Comparative characteristics of groups and assessment of systematic attrition error.

Indicator	Patients with Known Long-Term Outcomes (*n* = 693)	Patients with Unknown Long-Term Outcomes (*n* = 328)	*p*-Value
**Main clinical and anamnestic characteristics**			
Men, *n* (%)	547 (78.9)	238 (72.6)	0.024
Women, *n* (%)	146 (21.1)	90 (27.4)	0.024
Age, years, Me [Q1; Q3]	63.4 [57.8; 68.0]	63.3 [58.3; 68.0]	0.888
BMI, kg/m^2^, Me [Q1; Q3]	27.4 [24.6; 30.1]	27.7 [25.0; 30.9]	0.277
Obesity, *n* (%)	185 (26.7)	99 (30.2)	0.238
Type 2 DM, *n* (%)	171 (24.7)	113 (34.5)	0.001
• Newly diagnosed T2DM before CABG, *n* (%)	38 (5.5)	25 (7.6)	0.188
• Previously diagnosed T2DM, *n* (%)	133 (19.2)	88 (26.8)	0.006
Duration of T2DM, years, Me [Q1; Q3]	7.5 [4.0; 13.0]	5.0 [2.0; 8.0]	0.118
Prediabetes before CABG, *n* (%)	229 (33.0)	95 (29.0)	0.188
Any glucose metabolism disorder before CABG, *n* (%)	400 (57.7)	208 (63.4)	0.077
Arterial hypertension, *n* (%)	361 (52.1)	224 (68.3)	<0.001
III–IV FC angina, *n* (%)	154 (22.2)	21 (6.4)	<0.001
III FC CHF according to NYHA, *n* (%)	35 (5.0)	4 (1.2)	0.003
Unstable angina, *n* (%)	12 (1.7)	39 (11.9)	<0.001
Heart rhythm disturbances, *n* (%)	184 (26.5)	89 (27.1)	0.842
History of myocardial infarction, *n* (%)	254 (36.7)	129 (39.3)	0.412
History of stroke, *n* (%)	27 (3.9)	18 (5.5)	0.249
History of PCI, *n* (%)	64 (9.2)	49 (14.9)	0.006
History of CABG, *n* (%)	2 (0.3)	1 (0.3)	0.926
Smoking at the time of CABG, *n* (%)	107 (15.4)	47 (14.3)	0.643
Chronic kidney disease, *n* (%)	60 (8.7)	31 (9.5)	0.675
Intermittent claudication, *n* (%)	22 (3.2)	24 (7.3)	0.003
Lesion of 3 vessels according to coronary angiography, *n* (%)	318 (45.9)	153 (46.6)	0.823
Stenosis of the left main coronary artery ≥ 50%, *n* (%)	121 (17.5)	60 (18.3)	0.323
Carotid artery stenosis ≥ 50%, *n* (%)	202 (29.2)	58 (17.7)	<0.001
**Surgical characteristics**			
On-pump CABG, *n* (%)	591 (85.3)	323 (98.5)	<0.001
Off-pump CABG, *n* (%)	102 (14.7)	5 (1.5)	<0.001
Isolated CABG, *n* (%)	646 (93.2)	302 (92.1)	0.71
Combined operations, *n* (%)	47 (6.8)	26 (7.9)	0.41
• Carotid endarterectomy, *n* (%)	5 (0.7)	2 (0.6)	0.837
• Ventriculoplasty, *n* (%)	26 (3.8)	12 (3.7)	0.928
• Radiofrequency ablation, *n* (%)	2 (0.3)	3 (0.9)	0.2
• Mitral valve surgery, *n* (%)	4 (0.6)	5 (1.5)	0.14
• Aortic valve surgery, *n* (%)	3 (0.4)	1 (0.3)	0.778
• Thrombectomy, *n* (%)	7 (1.0)	3 (0.9)	0.884
Duration of operation, h, Me [Q1; Q3]	3.4 [3.2; 4.2]	3.5 [3.1; 4.2]	0.687
Days of hospital stay after CABG, Me [Q1; Q3]	14.0 [12.0; 17.0]	14.0 [12.0; 17.5]	0.209
**Preoperative medications, *n* (%)**			
• ACE inhibitors	489 (70.6)	241 (73.5)	0.326
• ARB (ARA II)	198 (28.6)	90 (27.4)	0.708
• β-blockers	626 (90.3)	286 (87.2)	0.134
• Calcium channel blockers	428 (61.8)	215 (65.5)	0.237
• Loop diuretics	287 (41.4)	151 (46.0)	0.163
• Thiazide diuretics			
• Statins	624 (90.0)	284 (86.6)	0.085
• Mineralocorticoid receptor antagonists	329 (47.5)	150 (45.7)	0.596
**Preoperative drug therapy for DM**	*n* = 171	*n* = 113	*p*-value
• Oral glucose-lowering agents, *n* (%)	146 (85.4)	70 (61.9)	<0.001
• Metformin, *n* (%)	82 (48.0)	52 (46.0)	0.747
• Sulfonylureas, *n* (%)	61 (35.7)	76 (67.3)	<0.001
• DPP-4 inhibitors, *n* (%)	7 (4.1)	4 (3.5)	0.806
• SGLT-2 inhibitors, *n* (%)	5 (2.9)	4 (3.5)	0.776
• GLP-1 agonists, *n* (%)	2 (1.2)	1 (0.9)	0.836
• Prehospital insulin, *n* (%)	39 (22.8)	3 (2.7)	<0.001

Notes: BMI—body mass index; CABG—coronary artery bypass grafting; CHF—chronic heart failure; DM—diabetes mellitus; DPP-4—dipeptidyl peptidase-4; FC—functional class; GLP-1—glucagon-like peptide-1; MI—myocardial infarction; NYHA—New York Heart Association; PCI—percutaneous coronary intervention; SGLT-2—sodium–glucose cotransporter-2; T2DM—type 2 diabetes mellitus.

## Data Availability

The original contributions presented in this study are included in the article. Further inquiries can be directed to the corresponding author.
